# A tip-coupled, two-cantilever, non-resonant microsystem for direct measurement of liquid viscosity

**DOI:** 10.1038/s41378-023-00483-6

**Published:** 2023-03-23

**Authors:** Sudhanshu Tiwari, Ajay Dangi, Rudra Pratap

**Affiliations:** 1 Center for Nano Science and Engineering, Indian Institute of Science, Bangalore, Karnataka 47906 India; 2 Department of Biomedical Engineering, Pennsylvania State University, University Park, PA 16802 USA; 3 Plaksha University, Mohali, Punjab, India; 4 Present Address: Oxide MEMS Lab, Purdue University, West Lafayette, USA

**Keywords:** Nanometrology, Electrical and electronic engineering

## Abstract

We report a non-resonant piezoelectric microelectromechanical cantilever system for the measurement of liquid viscosity. The system consists of two PiezoMEMS cantilevers in-line, with their free ends facing each other. The system is immersed in the fluid under test for viscosity measurement. One of the cantilevers is actuated using the embedded piezoelectric thin film to oscillate at a pre-selected non-resonant frequency. The second cantilever, the passive one, starts to oscillate due to the fluid-mediated energy transfer. The relative response of the passive cantilever is used as the metric for the fluid’s kinematic viscosity. The fabricated cantilevers are tested as viscosity sensors by carrying out experiments in fluids with different viscosities. The viscometer can measure viscosity at a single frequency of choice, and hence some important considerations for frequency selection are discussed. A discussion on the energy coupling between the active and the passive cantilevers is presented. The novel PiezoMEMS viscometer architecture proposed in this work will overcome several challenges faced by state-of-the-art resonance MEMS viscometers, by enabling faster and direct measurement, straightforward calibration, and the possibility of shear rate-dependent viscosity measurement.

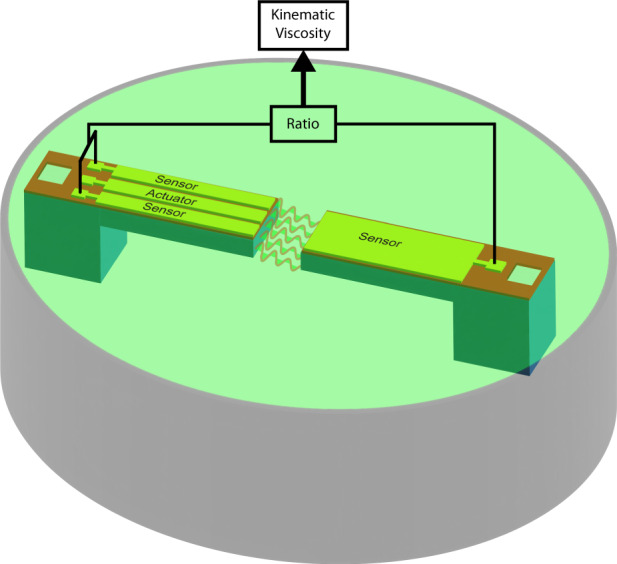

## Introduction

At a fixed temperature and pressure, the mass density and the viscosity have unique values for a given fluid. Accurate characterisation of fluids using these properties is of immense importance in healthcare, processing industries, and liquid (lubricant) health monitoring. For example, in healthcare applications, the viscosity of blood plasma can be a marker for several conditions such as cardiovascular diseases and autoimmune disorders like rheumatoid arthritis^1,2^. Elevated viscosity of whole blood is also associated with diminishing renal functions^3,4^. The estimation of viscosity is also essential for monitoring the quality of lubricants during operation^5^. In chemical processing industries, the viscosity of the byproducts and the end product is used as a quality control measure. Currently, most of these applications utilise a rotating cylinder-based bulky instrument for viscosity sensing which is expensive, time-consuming, and incompatible with the paradigm of internet-of-things (IOT) based distributed and online sensing. The use of bulky equipment for fluid-property measurement are expensive, require large operating power, and are slower. There is a need for cheaper, low cost and in-line measurement techniques for accurate viscosity measurement without any post-processing. Moreover, there is much emphasis on the fourth industrial revolution (Industry 4.0) led by a large number of interconnected smart devices for self-monitoring^6^.

Micro-scale solutions have emerged in the last decade to overcome the above challenges because they offer low cost, small footprint, and on-site installation for monitoring many properties and parameters. Piezoelectric MEMS resonators have become popular devices for the measurement of fluid properties because of their excellent ability to operate at a reasonable quality factor in a fluidic environment as evident from several publications over the last few years^7–12^. Moreover, PiezoMEMS devices offer low-voltage operation and high electromechanical coupling and hence are suitable for such applications.

Most MEMS-based fluid-property sensors use a single resonator (such as a cantilever) and track its resonance frequency, *f*_*r*_, and quality factor, *Q*, to estimate the density and viscosity of the fluid^7–12^. The obtained values depend on the fluid density and viscosity through secondary parameters^13^,

1 $$\begin{array}{ll}{f}_{r}&=\frac{{f}_{vac}\sqrt{1-\frac{1}{2{Q}^{2}}}}{\sqrt{1+\frac{L{g}_{2}}{m}}}\\ Q&=\frac{2\pi \sqrt{1+{g}_{2}/m}}{L{g}_{1}/m}\end{array}$$

In Eq. (1), *g*_1_ and *g*_2_ are the secondary parameters which are a series expansion in terms of density, viscosity, and their products. For example, ${g}_{1}={C}_{1}\sqrt{{f}_{r}}\sqrt{\rho \mu }+{C}_{2}$ and ${g}_{2}={C}_{3}\rho +{C}_{4}\frac{\sqrt{\rho \mu }}{\sqrt{{f}_{r}}}$ were used by the previous reports^13^. This method is time-consuming and demands extensive post-processing of the experimental data. These single-cantilever systems suffer from large errors, particularly for viscosity measurement^13^, because changes in quality factor can be due to a complex combination of several factors including density, viscosity, acoustic damping, substrate damping, and temperature^14^. Moreover, the resonators used in the current viscometers are operated in higher-order modes to achieve a better *Q*-factor^15^, since higher modes have less energy dissipation^16^. However, at very high frequencies, viscoelasticity can affect the measurement results.

Another popular category of sensors for liquid property measurement is acoustic wave sensors. However, the response of the SAW-based sensors depends on the square root of the product of density and viscosity ($\sqrt{\mu \rho }$). Moreover, the high-frequency operation of these sensors brings in the effect of the viscoelasticity of fluids. This, in turn, results in further complications in the measurements. Furthermore, the shear horizontal (SH) mode SAW devices saturate at $\sqrt{\mu \rho }=2$ making the design more complicated^4^. To overcome these challenges associated with SAW-based sensors, flexural modes of cantilever devices are actively being explored^17^.

We report a unique tip-coupled two-cantilever (TCTC) sensing system where the kinematic viscosity of any fluid can be obtained directly from the output parameters (velocity/displacement) of the device. Such a measurement technique offers a quick and direct measurement of viscosity. The use of two cantilevers in the proposed tip-coupled configuration allows us to eliminate the need for measurement of the *Q*-factor and associated complexity in the estimation of viscosity.

## The design of TCTC micro-viscometer

**Fig. 1:**
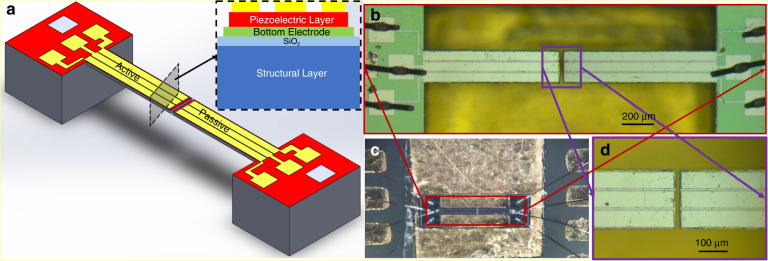
Viscosity measurement system using tow microcantilevers in a tip coupled arrangement. **a** Schematic representation of the design of the TCTC viscometer. **b** Optical image of the TCTC viscometer. **c** Optical image of the same device on a printed circuit board. **d** Magnified optical image of the system showing the gap between the two cantilevers

The design of the viscometer mimics a conventional rheometer/viscometer where the test fluid is subjected to shear between a fixed and a rotating object (plates or cylinders). In the conventional rheometer, the relationship between the applied torque and the velocity of the moving object is used as a measure of the liquid viscosity. The sensor design used in this study consists of two micro-cantilevers integrated with piezoelectric, lead zirconate titanate (PZT) thin film as the active element. Figure 1a shows the schematic representation of the design of the TCTC viscosity sensor. Figure 1b–d shows the top view of the fabricated TCTC structure at different magnifications. The cantilevers were fabricated on a 25-μm-thick silicon-on-insulator (SOI) wafer. Both the cantilevers are 200 μm wide. The active and passive cantilevers are 900 μm and 800 μm, respectively. The free ends of these cantilevers face each other with 20 μm between their faces. When the TCTC structure is placed in a fluid, the fluid takes the space between the cantilevers and acts as a coupling agent. In this case, the fluid is subjected to shear between the free ends of the two cantilevers. When an alternating electric field is applied across the piezoelectric film on the active cantilever, the cantilever starts vibrating at the frequency of the applied voltage. This sets the surrounding fluid in motion, which, in turn, applies a time-varying force on the passive cantilever and sets it in oscillatory motion. Since the passive cantilever is coupled to the active cantilever through the fluid medium, the amplitude of the vibration of the passive cantilever depends on the properties of the fluid and the vibration amplitude of the active cantilever. It is important to note that the fluid is subject to shear between the two cantilevers, and hence the effect of fluid viscosity dominates the response of the passive cantilever.

## Results

**Fig. 2:**
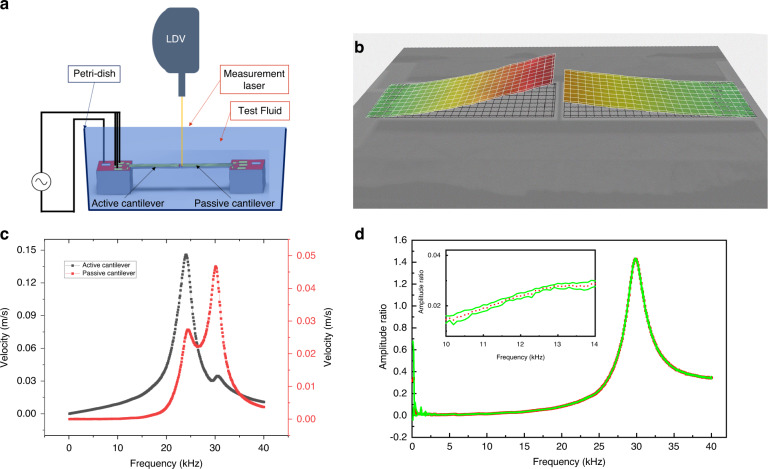
Measurement method and results of frequency responses of active and passive cantilevers. **a** A schematic representation of measurement of the frequency response of the cantilevers. **b** Measured deflection profile of active and passive cantilevers. **c** Frequency response of the active and the passive cantilever measured in 10% solution of glycerol and water. **d** The amplitude ratio (passive to active) in a 10% (by volume) solution of glycerol in water

Although split electrodes were designed and fabricated on the cantilevers for simultaneous actuation and sensing of the response, the sensor output was corrupted due to the capacitive feedthrough between actuation and sensing electrodes. Due to this difficulty, the cantilevers’ vibrational responses were measured using a laser Doppler vibrometer (LDV), Polytec MSA-400. The active cantilever was actuated by applying a unipolar electric potential of 0.75 V across the top (all three segments) and bottom electrodes of the PZT thin film while the sample was placed in a liquid (test fluid) filled petri-dish. The responses of the cantilevers (shown in Fig. 2c) were measured by placing the measurement laser beam on the tip of the cantilevers successively. The measurement setup is schematically shown in Fig. 2. The deflection profile of the cantilevers was also measured by scanning the whole cantilever system; the obtained profile is shown in Fig. 2b. Test fluid for which results are shown in Fig. 2 were obtained by preparing a 10% solution of glycerol in water. The solution’s corresponding density and kinematic viscosity values are 1026.9 kg/m^3^ and 2.59 cSt, respectively. The frequency response of each cantilever shows two peaks corresponding to the cantilevers’ resonance frequencies. We observe two peaks for each of the cantilevers because, at the respective resonance frequencies of the individual cantilevers, there is enhanced fluid movement, resulting in more fluidic coupling between the two cantilevers.

The responses of both cantilevers are affected by the properties of the fluid medium. When the device operates in a liquid of higher viscosity, the vibration amplitude of the cantilever reduces given the same actuation voltage. This variable response of the active cantilever results in variable coupling to the passive cantilever. In order to eliminate the variability of the coupling, we utilise the ratio of the amplitude of the passive cantilever to that of the active cantilever as the metric for viscosity measurement. The ratio of the responses is plotted in Fig. 2d for 10% solution of glycerol in water. This parameter is termed as “amplitude ratio, *R*” in rest of the manuscript. The amplitude ratio response has a peak at the resonance frequency of the passive cantilever, allowing the measurement of the resonance frequency of the passive cantilever immersed in the fluid using the same metric. This in turn can facilitate the estimation of the density of the fluid. The inset shown in Fig. 2d is the magnified view of the same plot in a narrower, 10–15 kHz frequency range. The red dots are mean values from three different measurements, and the green envelope bounds the error bar.

**Fig. 3:**
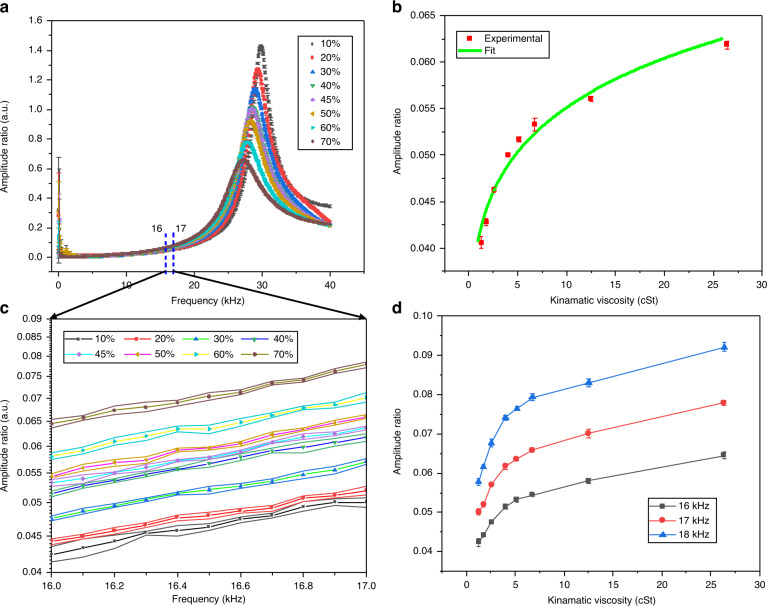
Effect of liquid viscosity on the amplitude ratio. **a** The amplitude ratios for varying concentrations of glycerol in water. **b** Zoomed-in view of the amplitude ratio vs frequency plot in the 16–17 kHz frequency range. The increase glycerol content is clearly discernible from the increase in the amplitude ratio. **c** Amplitude ratio vs kinematic viscosity plot for different concentrations of G–W solution. The kinematic viscosity values were obtained from the literature for the volumetric proportions used in the test samples. **d** Amplitude ratio vs kinematic viscosity plot for different concentrations of G–W solution at three different frequencies

The in-fluid measurements were repeated for different concentrations of glycerol–water (G–W) solutions. Figure 3a shows the ratio of the corresponding responses of the two cantilevers. Figure 3c shows the amplitude ratio vs frequency curve away from the resonance, between 16 and 17 kHz. This frequency range is selected based on the “Frequency Selection” logic discussed in the section “Frequency selection” of this paper. The increase in glycerol content is clearly discernible from the increasing amplitude ratio. In the very low-frequency region, the obtained data is noisy. The low-frequency noise can be attributed to Brownian noise^18^ and the low-frequency acoustic actuation of the cantilevers.

Figure 3b shows a plot of the amplitude ratio against the kinematic viscosity of the test fluids at 15.8 kHz. The values of the kinematic viscosity of G–W solution were taken from the literature^19–21^. A fit model, *aη*^*b*^ with the exponent *b* = 0.12, fits the experimental results. Such power law dependence results allow for a straightforward calibration of the sensor. This is considerably easier than the current resonant sensors where a Taylor series expansion of the hydrodynamic function is utilised for calibration requiring three or more experimental points^13^.

This method enables the viscosity measurement without the frequency sweep and the requirement of any peak fitting for *Q*-measurement, thereby enabling faster measurements. Moreover, a frequency sweep away from the resonance may provide information on the shear rate dependence of the viscosity of the fluid. Figure 3d shows the amplitude ratio vs kinematic viscosity plots at three different operating frequencies. Since the G–W mixture is a Newtonian fluid, the curves at higher frequencies have a higher amplitude ratio because of the variable sensitivity which is discussed in the next section.

## Frequency selection

Since the viscosity measurement can be carried out at a single actuation frequency, it is important to discuss the critical factors for the selection of the actuation frequency. The two most important considerations for frequency selection that are discussed in this section are, (i) sensitivity, and (ii) the penetration depth of the shear waves generated by the oscillating active cantilever.

### Sensitivity analysis

**Fig. 4:**
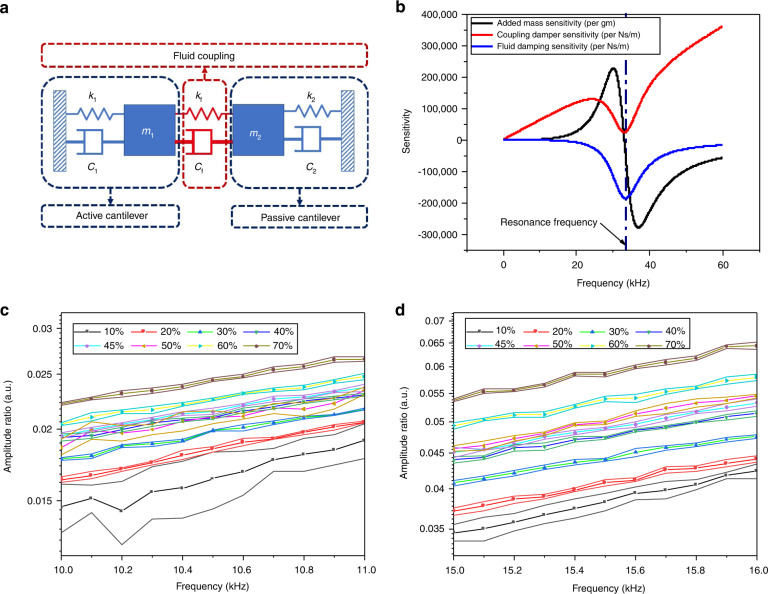
Modelling and sensitivity analysis of the cantilever system for viscosity measurement. **a** An illustrative model of the TCTC viscometer. The two cantilevers can be modelled as spring mass-damper systems and the fluid coupling can be modelled with a spring-damper system. For an incompressible flow of fluid, *k*_*f*_, can be ignored. **b** Sensitivity plots for added mass (black curve), coupling damper (red curve) and fluid damping (green curve). The plots suggest that the amplitude ratio in the low-frequency region is more sensitive to the change in the damping coefficient of the coupling damper in comparison to the changes in added mass and fluid damping. Resonance frequency is marked with the blue dashed line. **c** Experimental observation in a very low-frequency range. Small variations in viscosity are not discernible. **d** At a slightly higher frequency, the small variations in the viscosity could be observed through the amplitude ratio

The cantilever system can be modelled as a two-degree-of-freedom spring mass-damper system, as shown in Fig. 4a. The masses, *m*_1_ and *m*_2_ represent the effective masses of the active and passive cantilevers, respectively. The springs, *k*_1_ and *k*_2_ represent effective spring constants for the active and passive cantilevers, respectively. The dampers, *c*_1_ and *c*_2_ represent the damping coefficients associated with the vibration energy dissipation of each of the cantilevers. The fluid coupling can be incorporated in the model by introducing a coupling spring, *k*_*f*_ and a coupling damper, *c*_*f*_. For an incompressible flow of fluid, the presence of the coupling spring, *k*_*f*_ can be ignored. The damping coefficient *c*_*f*_ for the coupling damper depends on the fluid properties. Please refer to the Supplementary Materials for further details on the simplified model of this system.

To understand the effects of fluid density and viscosity on the amplitude ratio, the sensitivities of the amplitude ratio (*R*) were calculated and plotted for different parameters. Predominantly, fluid density contributes to the added mass and viscosity contributes to the damping. The mass sensitivity, $\frac{\partial }{\partial m}\left(R\right)$ provides information about the effect of the density of the fluid on the amplitude ratio. The coupling damper sensitivity, $\frac{\partial }{\partial {c}_{f}}\left(R\right)$ and the sensing cantilever’s damping coefficient sensitivity, $\frac{\partial }{\partial {c}_{2}}\left(R\right)$ provide some idea about the effect of the viscosity of the fluid on the measured amplitude ratio. Although the added mass sensitivity is calculated by differentiating the expression of, *R* with respect to *m*_2_, this sensitivity is valid for added mass also (the differential operator here is a linear operator). Specifically, $\frac{\partial }{\partial {c}_{f}}\left(R\right)$ provides information about the effect of fluid coupling between the active and the passive cantilevers. The green curve in Fig. 4b shows the sensitivity of the amplitude ratio with respect to change in the damping coefficient of the passive cantilever, (*c*_2_). The sensitivity is calculated for a cantilever exhibiting a resonance frequency of 34 kHz. It is well known that the response of the vibrating system is dominated by the damper only near the resonance frequency and hence we see that the amplitude ratio also follows the same trend.

Figure 4b shows a comparison between the sensitivities of the added mass and the coupling damper. The fluid density (added mass of the fluid) has a considerable effect on the frequency response of the cantilever near its resonance. In the low-frequency region, the effect of the added mass is negligible. The coupling damper sensitivity, first increases with frequency, then decreases near the resonance and increases again post the resonance. It is important to note that the response is sensitive to all three variables near the resonance. However, in the low-frequency region, the amplitude ratio is only sensitive to changes in the coupling damper. In the very low-frequency region, however, the sensitivity due to the coupling damper is also minimal. This behaviour is also visible in the experimental data, where the amplitude ratio is not discernible for small changes (40% to 50%) in the concentration of glycerol in water (see Fig. 4c), for the 10–11 kHz frequency range.

The clearly separated plots for 40–50% solutions (shown in Fig. 4d) in the slightly higher frequency range (15–16 kHz) also suggest that the sensitivity increases as we move to the higher frequencies. It is important to note that the low-frequency measurements also suffer from different noise sources such as acoustic noise and Brownian noise. The noise coupled with low sensitivity may result in undetectable changes (small) in kinematic viscosity.

Since we are interested in the measurement of viscosity through the coupling damper, we must choose the frequency in the region where the amplitude ratio is highly sensitive to the coupling damper, and is not sensitive to the added mass and fluid damping. Based on the sensitivity analysis presented above, we should be using the measurement results away from the resonance frequency (low-frequency region) for fluid-property measurement. However, the lowest frequency that can be used will be dictated by the noise-required sensitivity for that particular measurement. Fortunately, the sensitivity of the device can be varied (if the application demands) by just changing the operating frequency of the device and designing the resonance frequency of the cantilever.

### The penetration depth of shear waves

The transfer of energy from the active cantilever to the passive cantilever is through the shear waves generated by the active cantilever. The flow of shear waves created by an oscillating plate is governed by the characteristic length *δ*^22^,

$$\delta =\sqrt{\frac{2\eta }{\omega }}$$

where, *ω* is the frequency of oscillations and *η* is the kinematic viscosity of the fluid. The penetration depth of the shear waves follows an increasing trend with kinematic viscosity and a decreasing trend with frequency. Since the gap between the cantilevers is always fixed for a previously fabricated system, there can be an upper limit on the frequency and a lower limit on the viscosity.

#### Gap between the cantilevers

The concept of penetration depth also governs the gap between the cantilevers. Since there is an inverse relationship between the actuation frequency and the depth of penetration, as we increase the actuation frequency, the gap has to be reduced, to ensure effective coupling. The gap should be decided based on the minimum desired viscosity and maximum operational frequency.

## Performance metrics of the viscometer

### Sensitivity and range

**Fig. 5:**
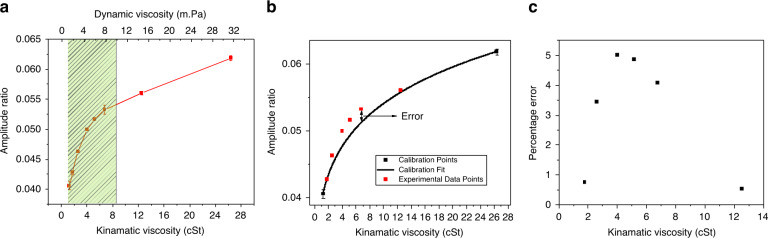
Target viscosity range and calibration error in the TCTC viscosity measurement system. **a** Amplitude ratio vs viscosity plot with the highlighted region of high sensitivity for target applications as shown in Table 1. **b** Expected curve of viscosity dependence when taking the two extreme experimental points as calibration points. The same plot also shows other experimental data points following the calibration curve. **c** Calibration error if the sensor is calibrated only two data points

The amplitude ratio, which is the metric for viscosity measurement for TCTC viscometer, follows an *ax*^*b*^ (with *b* < 1) dependence on kinematic viscosity. Such nonlinear dependence results in variable sensitivity in different measurement ranges. Since the exponent term in viscosity dependence is always smaller than unity, the sensitivity will follow a strongly decaying $\left(ba\times \frac{1}{{x}^{1-b}}\right)$ trend with viscosity. However, it is important to note that the amplitude ratio is a quantity calculated from the measured amplitudes of the active and passive cantilevers. Hence, the minimum discernible change in the viscosity will also be governed by the precision of the amplitude measurement and associated noise. The range of viscosity for several target applications is shown in Table 1. The viscosity range for these applications falls in the high sensitivity range of the current viscometer. The range of these targeted applications is also highlighted on the experimental curve of the viscometer operation as shown in Fig. 5a.

**Table 1:** Range of viscosity values for different applications

Target application	Viscosity range (mPa)	Reference
Study of bio-fluids	0.5-10	^23,24^
Monitoring of wine fermentation	1.4-2.4	^7,25^
Oils, paints, and varnishes	0.1-10	^24^

### Calibration error and precision

Since the experimental curve follows a power law *ax*^*b*^, the sensor can be calibrated by performing two calibration experiments to obtain the two coefficients “a” and “b”. The precision of the sensor will be governed by the ability of these coefficients to capture the true viscosity values for other experiments. The expected curve for the dependence of the amplitude ratio on the kinematic viscosity of the fluid is shown in Fig. 5b (solid black curve). This curve was generated by selecting two significantly different viscosities as calibration points. The same plot also shows the experimental data points and the deviation of these data points from the calibrated curve. The maximum error due to calibration between different experimental points is ~5% (see Fig. 5c). As explained previously, the resonant sensors are calibrated by evaluating the constants of a series expansion of the hydrodynamic function. The best reported maximum error for such a resonant sensor, calibrated using four constants at three different temperatures (effectively running 12 calibration experiments) is 7%^13^.

## Coupling mechanisms

There are three modes of energy transfer from the active cantilever to the passive cantilever, namely structural coupling, coupling due to fluid flow, and acoustic coupling. The structural coupling has no effect on the viscosity measurement because it just adds a constant bias in the measured response. Both the acoustic coupling and the viscous coupling, affect the response of the passive cantilever. Moreover, both of these coupling mechanisms are affected by the kinematic viscosity of the fluid medium.

From a basic Stokes-flow assumption, the force on the passive cantilever due to the oscillations of the active cantilever should follow a $a\sqrt{\eta }$ dependence^22^. The acoustic pressure wave, on the other hand, follows an exponentially decaying curve. The origin of these expressions is explained in the Supplementary Material. In order to verify this behaviour, the experimental data were fitted to the following curve,

$$R=a\sqrt{\eta }+b{e}^{-c\times \eta }+d$$

**Fig. 6:**
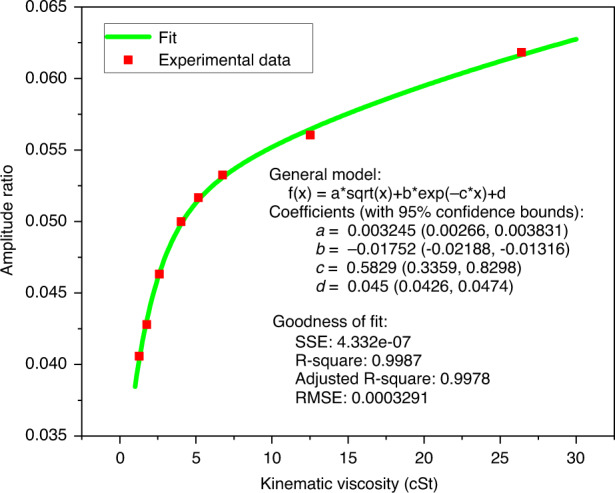
The experimental data fitted with the expression $R=a\sqrt{\eta }+b{e}^{-c\times \eta }+d$. The square-toot term is the result of viscous flow, the exponentially decaying term comes from the acoustic coupling and the constant term signifies the structural coupling

The corresponding fit along with the experimental data points are plotted in Fig. 6. The fit parameters and the goodness of fit are also given in the same plot. The near-perfect fit is evidence of the presence of all three modes of energy coupling, described above.

## Conclusions

The TCTC viscometer design presented in this paper utilises a novel two-cantilever architecture, where the fluid coupling between the tips of the cantilevers is exploited for viscosity sensing. This unique approach is a significant improvement over the current MEMS viscosity sensors, since it enables a direct, faster, and potentially, more sensitive measurement. The relative response of the passive cantilever follows a power law, *aη*^*b*^ dependence on the kinematic viscosity of the fluid. Such a dependence allows for calibrations of the sensor using only *two* data points as opposed to four or more in the current MEMS viscometer. Another significant advantage of the TCTC viscometer is its ability to measure the viscosity of the liquid at different operating frequencies thereby allowing shear rate-dependent measurement. Although the test fluid used in this paper is a Newtonian fluid, the viscometer’s ability to measure viscosity at different frequencies is explained. We believe these initial results are encouraging for the MEMS community to explore the avenues of viscometry using TCTC design.

In this paper, we have also presented an understanding of the energy coupling mechanism between the two cantilevers. It is proposed that in addition to the shear flow of fluid, the energy transfer between the cantilevers can also be due to the structural coupling (originating because the two cantilevers share a common frame) and due to the flow of acoustic waves. This hypothesis has been validated by fitting the experimental data to the appropriate expression which includes all three coupling mechanisms.

## Supplementary information

Supplementary Material
