# Supplementary material for: A tip-coupled, two-cantilever, non-resonant microsystem for direct measurement of liquid viscosity
Source: Microsyst Nanoeng. 2023 Mar 23;9:34. doi: 10.1038/s41378-023-00483-6 (PMC10033823; doi:10.1038/s41378-023-00483-6)
Supplement: Supplementary file 1 — Supplementary Material [file 41378_2023_483_MOESM1_ESM.pdf]

Supplementary Material for the Manuscript Titled, "Tip-Coupled, Two-Cantilever, Non-resonant Microsystem for Direct Measurement of Liquid Viscosity"

March 8, 2023

1 Fabrication of Cantilever System

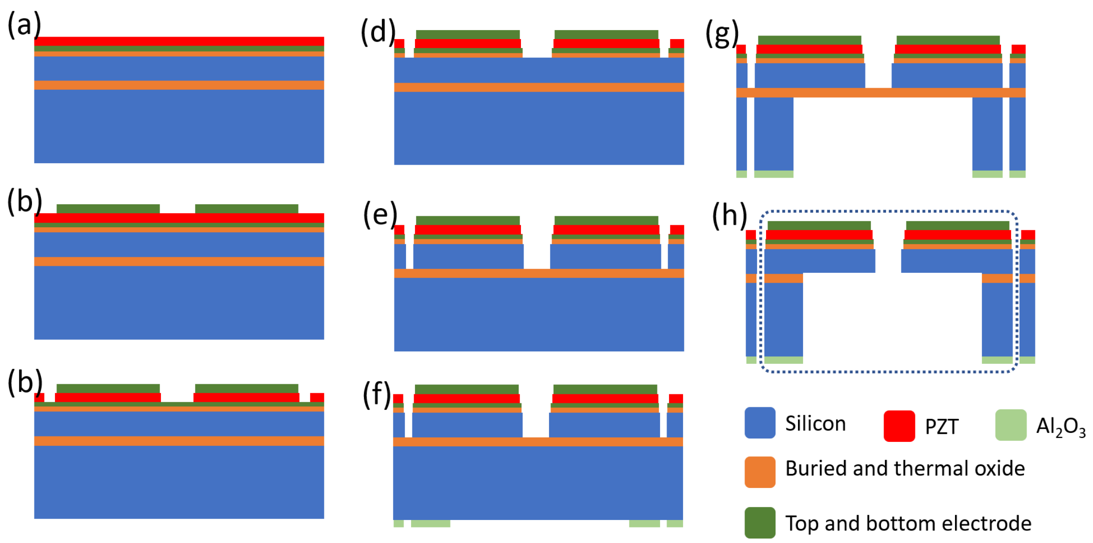

Figure S1: The process flow used for the fabrication of the TCTC micro-viscometer. (a) Starting material stack: PZT on a SOI wafer. (b) Patterned deposition of top electrodes through lift-ff process. (c) Wet patterning of PZT thin film. (d) Patterning of bottom electrode (Pt) and thermal oxide. (e) Patterning of device layer of SOI wafer using DRIE. (f) Deposition and patterning of thin Al_2O_3 layer as mask for bottom side DRIE. (g) Patterning of handle layer of SOI wafer. (h) Removal of buried oxide using RIE to release the structure.

The devices used in this study were fabricated using a standard fabrication process developed by the authors. While a brief overview of fabrication method is given here (see Figure S1), the details of fabrication methodology can be found in earlier reports[3, 4]. A PZT coated SOI wafer with 25 μm device layer thickness is used for fabrication of the cantilevers. A patterned top electrode of platinum (Pt) thin film with titanium (Ti) adhesion layer is achieved via lift-off process after sputter deposition. Both PZT and Pt bottom electrodes were patterned using separate mask and different recipes. The thermal oxide is

patterned using reactive ion etching (RIE) and the device layer of the SOI is patterned using deep RIE (DRIE) method. The sample is then processed from backside to remove the handle layer of SOI, again using DRIE. A thin Al_2O_3 layer is used as the mask for backside etching in DRIE. Finally, the buried oxide is removed in an RIE tool to release the devices.

2 Expressions for Sensitivity Analysis

The equivalent 2-DOF model for the viscometer is shown in Figure 4(a) of the manuscript. For a given force, the frequency responses of the masses m_1 (denoted as x_1) and m_2 (denoted as x_2) are shown in eqn. (1). The individual responses of both the masses depend on the coupling damper and the fluid damping. To reduce the number of unknowns, a metric R which is the ratio of the responses of the masses m_2 and m_1 is chosen. The expression for R shown in eqn. (1). Since the ratio only depends on the properties of the fluid (through coupling damper) and the parameters related to passive cantilever, we can eliminate the effect of change in response of the active cantilever from viscosity measurement.

$$\begin{aligned} x_1 &= \frac{\sqrt{\left(c_f + c_2\right)^2 \omega^2 + \left(k_2 - m_2\omega^2\right)^2}}{\sqrt{A^2+B^2}} \\ x_2 &= \frac{\sqrt{c_f^2\omega^2}}{\sqrt{A^2+B^2}} \\ R = \frac{x_2}{x_1} &= \frac{\sqrt{c_f^2\omega^2}}{\sqrt{\left(c_f + c_2\right)^2 \omega^2 + \left(k_2 - m_2\omega^2\right)^2}} \end{aligned} \tag{1}$$

where,

$$\begin{aligned} A &= \left(c_f + c_2\right)\omega\left(k_1 - m_1\omega^2\right) + \left(c_1 + c_f\right)\omega\left(k_2 - m_2\omega^2\right) \\ B &= c_f^2\omega^2 - \left(c_1 + c_f\right)\left(c_f + c_2\right)\omega^2 + \left(k_1 - m_1\omega^2\right)\left(k_2 - m_2\omega^2\right) \end{aligned}$$

By differentiating the expression of R (given in eqn. 1) with respect to m_2, c_f, and c_2, we can obtain the sensitivities of amplitude ratio for a small change is added mass, kinematic viscosity (through coupling damper), and fluid damping respectively.

$$\begin{aligned} \frac{\partial}{\partial m_2}(R) &= \frac{\omega^2\sqrt{\omega^2 c_f^2 + k_f^2}\left(k_f + k_2 - m_2\omega^2\right)}{\left(\omega^2\left(c_f + c_2\right)^2 + \left(k_f + k_2 - m_2\omega^2\right)^2\right)^{3/2}} \\ \frac{\partial}{\partial C_f}(R) &= \frac{\omega^2\left(c_f + c_2\right)\sqrt{\omega^2 c_f^2 + k_f^2}}{\left(\omega^2\left(c_f + c_2\right)^2 + \left(k_f + k_2 - m_2\omega^2\right)^2\right)^{3/2}} \\ \frac{\partial}{\partial C_2}(R) &= \frac{\omega^2\left(c_f\left(k_2 - m_2\omega^2\right)\left(2k_f + k_2 - m_2\omega^2\right) + c_2\left(\omega^2 c_f^2 - k_f^2\right) + c_2^2\omega^2 c_f\right)}{\sqrt{\omega^2 c_f^2 + k_f^2}\left(\omega^2\left(c_f + c_2\right)^2 + \left(k_f + k_2 - m_2\omega^2\right)^2\right)^{3/2}} \end{aligned}$$

3 Stokes-Couette Flow Equivalence

The operation of the viscometer can be modelled as a simplified Stokes-Couette flow which is a combination of stokes second problem and Couette flow. In Stokes second problem, an oscillating plate generates

a flow in an unbounded fluid domain while in Couette flow the fluid flows between two plates in response to the movement of one plate. The Stokes-Couette flow combines the two flows such that the fluid flows between two plates in response to the oscillatory motion of one of the plates while the other plate is fixed. The equivalent model of the coupling damper between the two masses can be derived from forces on the fixed wall during a Stokes-Couette flow. We assume a scenario where a damper is attached between a moving mass and a fixed wall, as shown in Figure S2, which is equivalent to the perfect Stokes-Couette flow. The force on the fixed wall because of the movement of the mass is given as $c_f \dot{x}$, where $\dot{x}$ is the velocity of the moving mass. For the Stokes-Couette flow with one wall moving with a velocity $u = u_0 e^{i\omega t}$, the force on the fixed wall is given as the real part of the expression[1] shown bellow,

$$F = \frac{\sqrt{\omega\eta}\,(1+i)\,u\csc\left(\left(\sqrt{\frac{\omega}{2\eta}}\right)(1+i)\,d\right)}{2} \tag{2}$$

where η is the kinematic viscosity of the fluid which is the ratio of the dynamic viscosity μ and the mass density ρ of the fluid, ω is the frequency of oscillation of the moving plate, and d is the gap between the two plates.

The force on the second plate is proportional to $\sqrt{\omega\eta}$. Similarly, for a damper connected between a fixed wall and a moving mass, the force on the fixed wall is proportional to the damping coefficient C. By the analogous model, as illustrated in Figure S2 the damping coefficient (C) of the damper can be assumed to be proportional to the $\sqrt{\omega\eta}$ term. For a fixed frequency of oscillations the damping coefficient is proportional to the square root of the kinematic viscosity.

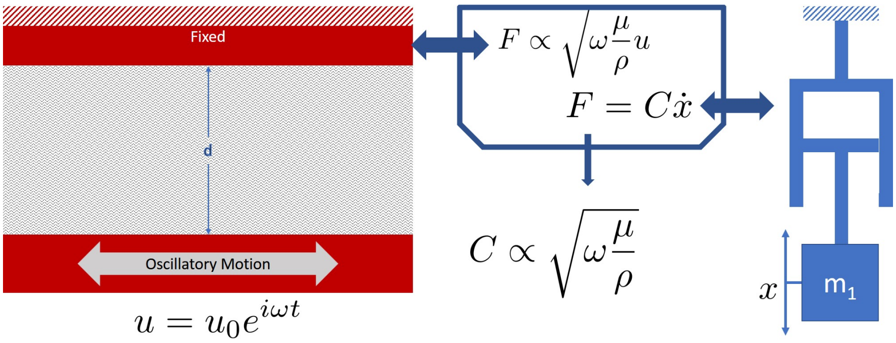

Figure S2: An schematic representation of the modelling of the coupling damper for the Stokes-Couette flow.

4 Acoustic Coupling

An oscillating body inside a fluid medium generates sound (acoustic) waves. The propagation of such pressure waves follows the well known stokes law of sound attenuation [2]. As the sound waves propagates away from the source the amplitude of the sound pressure decreases exponentially with distance (d) as shown bellow,

$$P_d = P_0 \exp\left(\frac{2\eta\omega^2}{3V^3}d\right)$$

where, P_d is the sound pressure at a distance d from the source, P_0 is sound pressre near the source, η is the kinematic viscosity of the medium, ω is the frequency, V is speed of sound in that medium.

It is important to note that for a fixed frequency and at given distance from the source the sound pressure decreases exponentially with the kinematic viscosity of the fluid. Hence, in case of of our TCTC system, the coupling due to flow of acoustic energy should also follow the exponentially decaying trend.

5 Effective Coupling

In addition to the two coupling mechanisms explained above, there can be some structural coupling between the two cantilevers because the two cantilevers are supported by a frame. Combining these three effects together we suggest that the ratio of amplitudes of passive to active cantilever should follow the expression sown in eqn. 3.

$$R = a\sqrt{\eta} + be^{-c\times\eta} + d \tag{3}$$

References

[1] L D Landau and E M Lifshitz. *Fluid Mechanics*. Second Edi, pp. 83–88.

[2] "On the Theories of the Internal Friction of Fluids in Motion, and of the Equilibrium and Motion of Elastic Solids by G. G. Stokes, M.A., Fellow of Pembroke College". In: *Classics of Elastic Wave Theory*. 2013, pp. 125–162. DOI: 10.1190/1.9781560801931.ch3e. URL: https://library.seg.org/doi/abs/10.1190/1.9781560801931.ch3e.

[3] Sudhanshu Tiwari et al. "Enabling Fabrication of PZT Based PiezoMEMS Devices". In: *IEEE Sensors 2018*. IEEE, 2018.

[4] Sudhanshu Tiwari et al. "Low cost, contamination-free, and damage-free fabrication of PZT MEMS on SOI substrate". In: *Journal of Micromechanics and Microengineering* 32.2 (2021), p. 025003.
